# Comparison of surgical efficacy of locking plates and interlocking intramedullary nails in the treatment of proximal humerus fractures

**DOI:** 10.1186/s13018-022-03360-6

**Published:** 2022-11-05

**Authors:** Zheming Guo, Linchao Sang, Qingting Meng, Lijun Tian, Yingchao Yin

**Affiliations:** 1 Department of Orthopaedic Surgery, The Third Hospital of Shijiazhuang City, Shijiazhuang, China; 2 Department of Orthopaedic Surgery, Third Hospital of Hebei Medical University, Shijiazhuang, China

**Keywords:** Proximal humeral fracture, Locking plate, Interlocking intramedullary nail, Clinical efficacy

## Abstract

**Background:**

The objective of this study was to evaluate the efficacy of locking plates versus interlocking intramedullary nails in the treatment of proximal humerus fractures to provide clinical data support and theoretical guidance.

**Methods:**

Patients with proximal humerus fracture from the Third hospital of Shijiazhuang city and Third hospital of Hebei medical university and from January 2017 to June 2019 were selected, included and divided into the locking plate group and the interlocking intramedullary nail group according to the intervention received. Information pertaining to the perioperative period (operation time, hospital stay, blood loss, etc.) of patients in both groups was collected. VAS pain scores, shoulder activity Constant-Murley scores and postoperative complications were documented. The perioperative data of the two groups were compared, and *P* < 0.05 was considered statistically significant.

**Results:**

A total of 64 patients were enrolled, including 36 patients in the locking plate group, with a mean age of 61.3 ± 13.9 years, while the mean age of the interlocking intramedullary nail group was 65.6 ± 11.2 years. There was no statistical difference in gender, affected side, injury mechanism and Neer classification between the two groups (*P* > 0.05). However, the average operation time of the locking plate group was shorter than that of the interlocking nail group (84.9 ± 11.7 vs. 102.6 ± 22.1 min, *P* = 0.00), and the intraoperative blood loss of the locking plate group (137.4 ± 16.8 ml) was higher than that of the interlocking nail group (72.5 ± 10.5 ml, *P* = 0.00). There was no significant difference in the VAS score and Constant-Murley score between these two groups at the final follow-up.

**Conclusion:**

Interlocking intramedullary nails are more minimally invasive than locking plates, but fracture reduction and fixation take longer. There was no significant difference in pain and shoulder function scores between the two internal fixation strategies for the treatment of proximal humerus fracture.

## Introduction

Proximal humerus fractures (PHFs) are common fragility fractures in the aging population, occupying about 4–5% of all fractures [1]. The choice of treatment for PHFs remains controversial, especially in osteoporotic and elderly patients [2]. The comorbidity, poor bone quality and degeneration of rotator cuff will affect the outcome of treatment. Non-operative treatments might benefit non-displaced or slightly displaced PHFs, proving good clinical effects [3]. While, as the population ages, the proportion of non-displaced proximal humerus fractures has declined from 85 to 42% [4]. Surgical reduction and internal fixation might be needed for the displaced cases, including two-, three- and four-part of PHFs [5]. How to protect the blood supply of the humeral head while restoring the bone alignment and articular surface flatness is a challenging problem.

Surgical strategies for PHFs have gone through a long evolution over the last few decades. The most widely used treatment include open reduction and internal fixation with locking plate osteosynthesis, intramedullary nail fixation, reverse shoulder arthroplasty and shoulder hemiarthroplasty. There is still controversy surrounding the treatment approaches used in displaced PHFs. The locking plate strategy is considered the gold standard treatment for PHFs, but several studies reported an association with several complications [6]. The placement of the locking plate requires dissection of the extensive muscles, which might damage the nourishing vessels to the bone and lead to nonunion or necrosis [7]. There is also an increased risk of injury to the axillary nerve if the plate is inserted applying the percutaneous technique [8]. There are several biomechanical advantages to using intramedullary nailing to fix PHFs, including higher lateral and torsional stress stiffness. However, there is a risk of rotator cuff tear [9], which may lead to functional limitation or shoulder pain.

The aim of this retrospective study was to analyze these two methods, provide clinically referable evidence on the clinical outcomes, and report the complications related to each intervention.

## Materials and methods

### Study design and participants

We retrospectively analyzed all the patients diagnosed with PHFs between January 2017 and June 2019 in the Third hospital of Hebei medical university and the Third Hospital of Shijiazhuang city. The inclusion criteria were as follows: (1) patients over 18 years of age; (2) two-part, three-part, or four-part of PHFs on Neer classification; (3) fresh fracture; (4) no previous ipsilateral humeral surgery. Meanwhile, the exclusion criteria were as follows: (1) pathologic fractures or open fractures; (2) concomitant neurovascular injury; (3) fractures associated with shoulder dislocation; (4) mental illness. The committee of our institution waived the requirement for written informed consent because this present study was retrospective.

### Surgical procedure

The patients were operated under brachial plexus block or general anesthesia. All surgeries were conducted by the same team, which was proficient in both techniques. All patients were lying on a radiolucent operating table in a beach chair position.

For the intramedullary nail group, the deltoid-splitting approach was applied. After exposing the deltoid muscle, the anterior and middle deltoid bundles were split bluntly along the direction of the muscle fibers to reach the fracture site. The entry point was revealed after temporary fixation of the fracture fragments with 2.0 mm Kirschner wires, which was 1 cm medial to the greater tuberosity. Then the Kirschner wire was used as a “joystick” to manipulate the humerus head and reduce the displacement. After the tuberosities were reduced, the three- or four-part fracture type would turn into a two-part type. Then the main nail (Targon nail) was inserted, ensuring that the end of the intramedullary nail was 2–3 mm below the cartilage of the humeral head. At last, the proximal and distal screws were locked.

For the locking plate group, the classical deltopectoral approach was applied. Both the indirect and direct reductions were performed to reduce the fracture fragments with the assistance of the C-arm. The bone defect of the humeral head was evaluated, and autologous or allogeneic iliac bone grafting was used for patients with poor bone quality and larger defects to increase the stability after plate fixation. At least five locking screws were inserted proximally, with a minimum of 3 distal screws. The nonabsorbable sutures passed through the holes on the plate (PHILOS, DePuy Synthes) and were knotted to repair the rotator cuff and increase its stability.

### Clinical and radiographical assessment

Clinical data and radiographic materials of all the included patients were collected, including gender, age, injury type, comorbidities, operation time, time to surgery, blood loss, and Neer classification [10]. The visual analog scale (VAS) for pain and the Constant-Murley Score was determined for shoulder function assessment.

### Postoperative management and rehabilitation protocol

Shoulders were immobilized for four weeks with an abduction pillow sling. Passive movement of the shoulder began on the second day after surgery. Active movements started 4–6 weeks postoperatively.

Clinical follow-up was conducted by two orthopedics attending physicians at 1 and 3 months after surgery. Postoperative plain radiographs were taken at each follow-up. Furthermore, all surgery-related complications were recorded, such as screw breakage, superficial infection, fat liquefaction, varus deformity, delayed union, and acromion impingement.

### Statistical analysis

All statistical analyses were performed using IBM SPSS Statistics for Windows, version 20.0 (SPSS, Inc., Chicago, IL, USA). The distributions of all variables were evaluated for normality using the Shapiro–Wilk test. Data that satisfies normality were presented as the mean ± standard deviation. Those data that did not meet normality were presented as medians and quartiles. Chi-squared test were used to analyze the difference in gender distribution, Neer classification of humeral head fractures and injury mechanism between the two groups. The nonparametric test and Student *t* test were applied to analyze continuous variables. A value of *P* less than 0.05 was considered statistically significant.

## Results

In total, 103 proximal humerus fracture patients were searched during this period (January 2017 to June 2019). All the patients were evaluated by two surgeons according to the inclusion and exclusion criteria. After screening, 38 patients were excluded. Sixty-five patients with locking plates and interlocking intramedullary nails were primarily included, 1 of whom was lost to follow-up. The demographic data of these two groups are displayed in Table 1. There was no statistical difference in gender, side, cause of injury and Neer classification between the two groups (*P* > 0.05). The average surgery time was shorter in the locking plate group compared to the intramedullary nail group (84.9 ± 11.7 ml vs. 102.6 ± 22.1 ml, *P* = 0.000). The blood loss was 137.4 ± 16.8 ml in the locking plate group, which was higher than in the intramedullary nail group (72.5 ± 10.5 ml, *P* = 0.000). There was no difference in the VAS score (*P* = 0.202) and Constant-Murley score (*P* = 0.067) at the final follow-up, as shown in Table 2.

**Table 1:** Comparison of patient baseline in the locking plate group versus the interlocking intramedullary nail group

	Locking plate group (*n* = 36)	Intramedullary nail group (*n* = 28)	*P* value
Gender			0.628
Male	15	10	
Female	21	18	
Affected side			0.728
Left	19	16	
Right	17	12	
Age (Years)	61.3 ± 13.9	65.6 ± 11.2	0.194
Injury mechanism			0.539
Fell down	20	19	
High fall injury	3	1	
Traffic accident	13	8	
Neer classification			0.519
Two-parts	13	11	
Three-parts	17	15	
Four-parts	6	2	
Surgery time (min)	84.9 ± 11.7	102.6 ± 22.1	0.000
Blood loss (ml)	137.4 ± 16.8	72.5 ± 10.5	0.000
Length of stay (day)	12.2 ± 4.1	10.6 ± 3.5	0.101
Follow-up time (month)	8.6 ± 1.3	9.2 ± 1.7	0.120

**Table 2:** Comparison of VAS and Constant-Murley scores between the locking plate group and the interlocking intramedullary nail group

Groups	1 month postoperatively		3 month postoperatively		Final follow-up	
	VAS score	Constant-Murley score	VAS score	Constant-Murley score	VAS score	Constant-Murley score
Locking plate group (*n* = 36)	4.5 ± 1.1	40.3 ± 7.2	2.1 ± 0.6	62.3 ± 5.5	0.5 ± 0.5	85.9 ± 6.7
Interlocking intramedullary nail group(*n* = 28)	4.2 ± 0.9	45.1 ± 9.6	1.8 ± 0.4	70.6 ± 7.9	0.6 ± 0.5	83.1 ± 5.3
*t*	1.010	− 2.273	2.379	− 4.758	1.289	1.863
*P* value	0.316	0.026	0.020	0.000	0.202	0.067

The incidence of general complications was 8.33% (3 patients) and 10.71% (3 patients) in the locking plate and intramedullary nail group, respectively (*P* = 0.343), as shown in Table 3. One patient developed a superficial infection, and one had fat liquefaction in the locking plate group. One patient developed varus deformity in the intramedullary nail group at the final follow-up. Two patients in the intramedullary nail group and one in the locking plate group had acromion impingement postoperatively.

**Table 3:** Postoperative complications in the locking plate group and the interlocking intramedullary nail group

	Locking plate group (*n* = 36)	Interlocking intramedullary nail group (*n* = 28)
Screw breakage	0	0
Superficial infection	1	0
Fat liquefaction	1	0
Varus deformity	0	1
Delayed union	0	0
Acromion impingement	1	2
χ^2^		3.333
*P* value		0.343

## Discussion

**Fig. 1:**
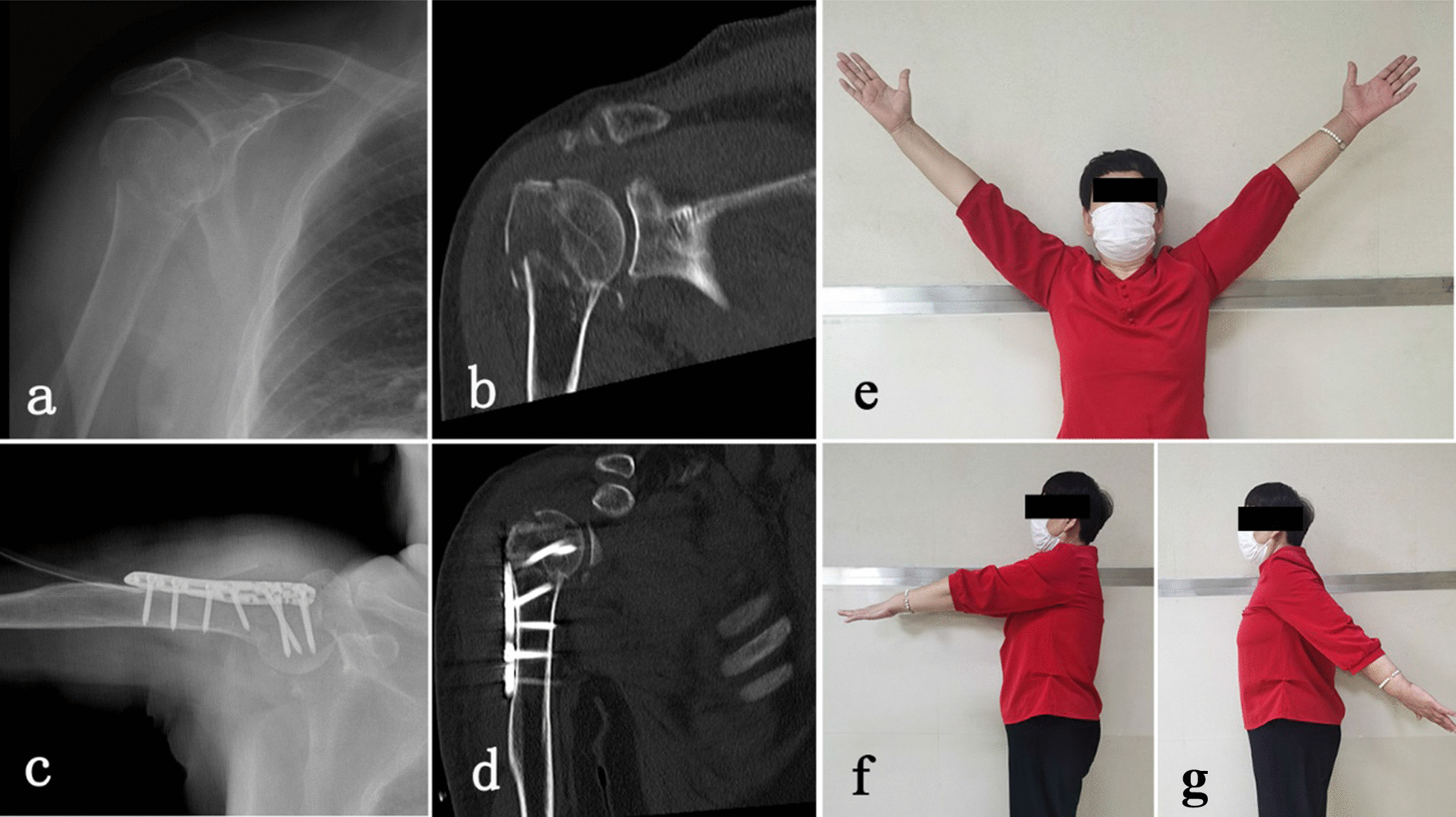
Patients with PHFs were treated using the locking plate approach. **a** Preoperative X-ray of the shoulder joint; **b** preoperative CT; **c** Postoperative X-ray; **d** Postoperative CT scan of the shoulder joint; **e**, **f**, **j** functional results at the final follow-up

**Fig.2:**
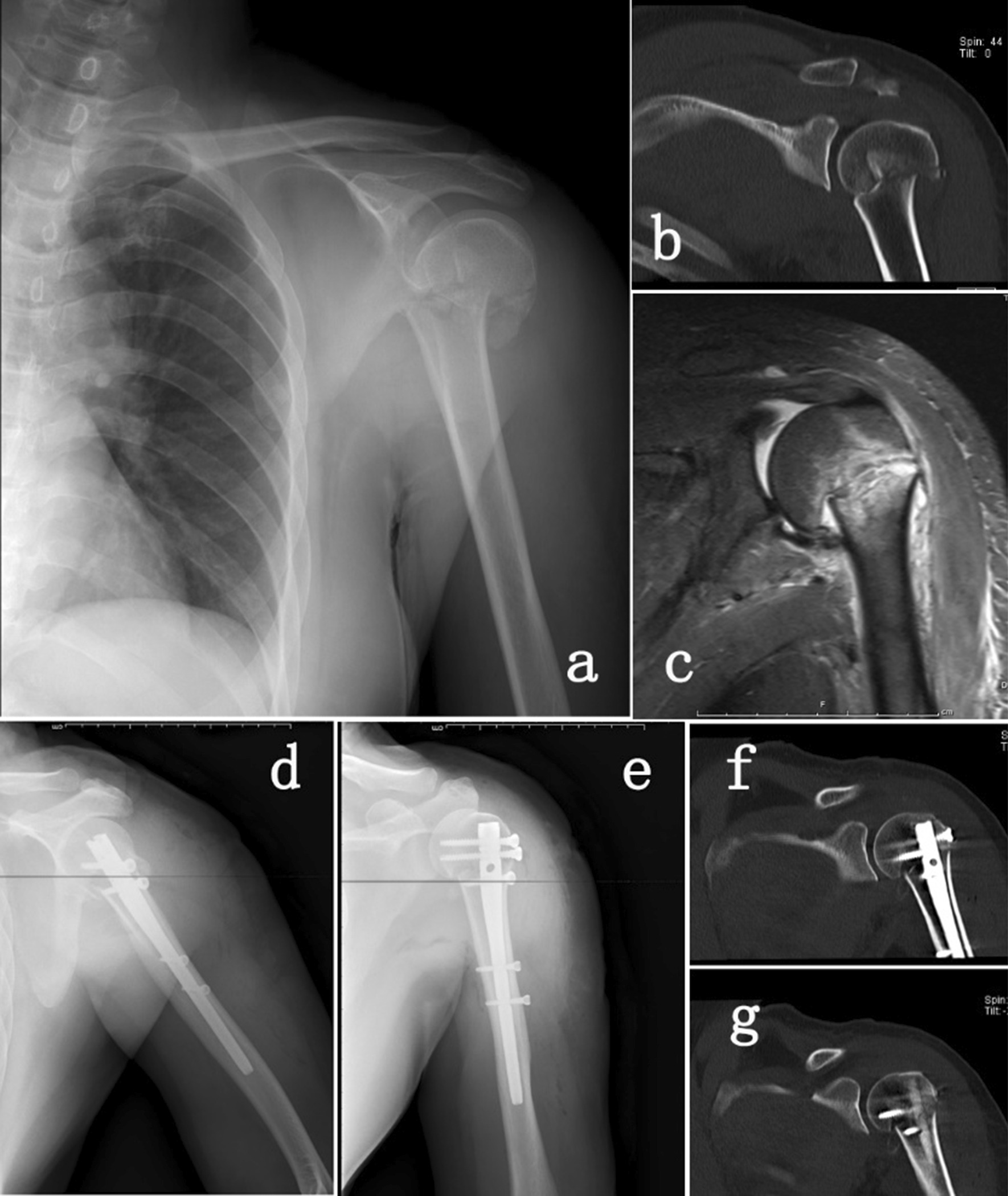
Patients with PHFs were treated with the interlocking intramedullary nail approach. **a** Preoperative X-ray of the shoulder joint; **b** preoperative CT; **c** Preoperative MRI; **d**, **e** Postoperative X-ray; **f**, **g** Postoperative CT scan of the shoulder joint

Herein, we performed a retrospective study comparing locking plates and intramedullary nails in the treatment of PHFs. Our findings show that the average operation time of the locking plate group was shorter than that of the interlocking nail group, and the intraoperative blood loss of the locking plate group was higher than that of the interlocking nail group. No significant difference was found in the VAS score and Constant-Murley score between the two groups at the final follow-up. (Figs. 1 and 2)

**Fig.3:**
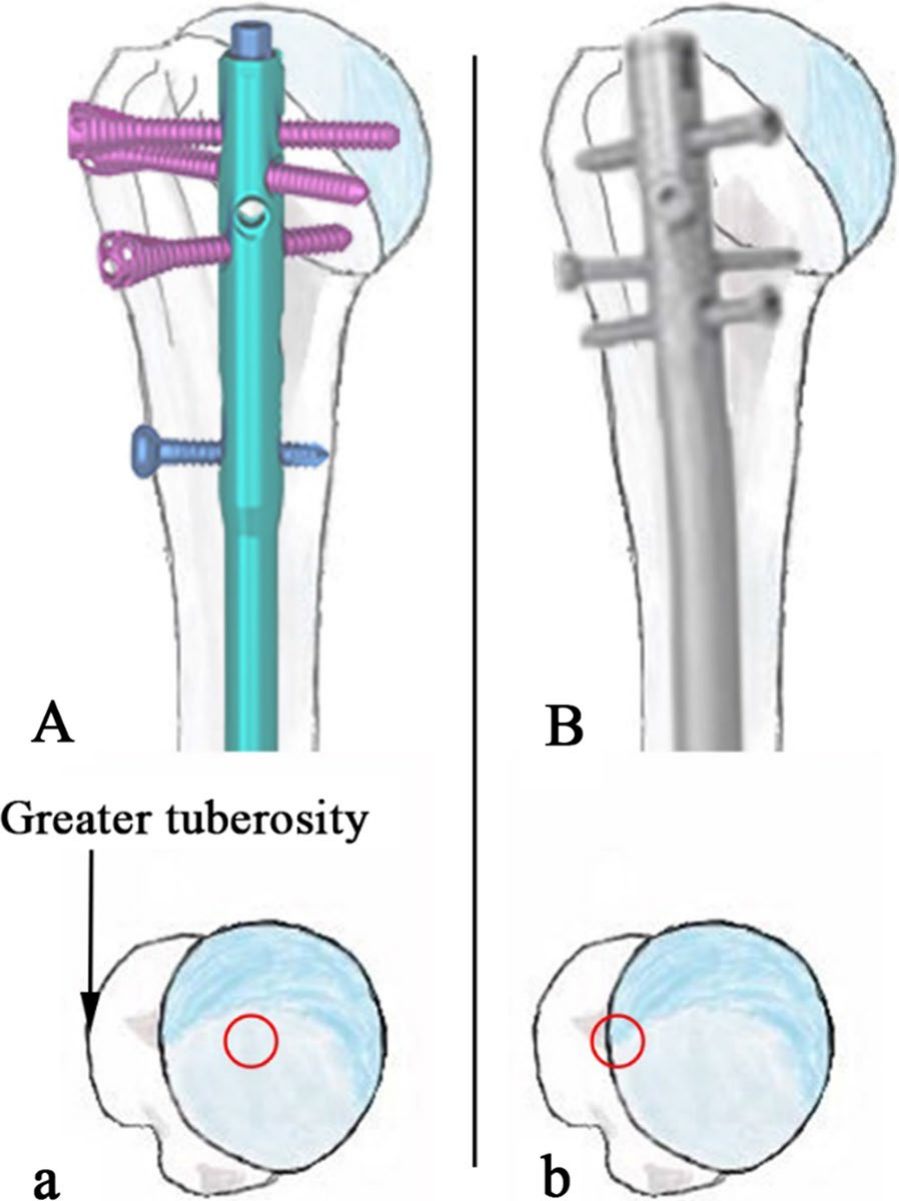
The entry point of the third generation intramedullary nail (straight nail) (**A, a**) is more medial than the second generation intramedullary nail (curved nail) (**B, b**)

Second-generation intramedullary nails (curved) were used in this trial. The entry point of the second-generation nail was more medial to the greater tuberosity (Fig. 3), which is comes with a higher risk of rotator cuff injury and shoulder pain [11–13]. Contrary to previous studies, no rotator cuff lesions were detected in this study. Placing the end of the main nail 2–3 mm below the cartilage of humeral head and repairing the rotator cuff with precision sutures after the operation may explain the absence of postoperative-related dysfunction. In this study no difference was found between the plate group and nail group in terms of VAS and Constant-Murley scores at the final follow-up. The straight intramedullary MultiLoc nail (Depuy Synthes) is a known third-generation intramedullary nail. Multiloc changed the design of the proximal 4–6° valgus angle of the second-generation intramedullary nail to that of a proximal straight nail [11, 14]. The straight design theoretically leaves a safe zone between the lateral fracture fragment and the nail entry hole in the humeral head to avoid an unpredictable crack in this area (Fig. 3). Meanwhile, the end of the nail can be embedded under the cartilage, which will reduce the risk of postoperative acromion impingement.

During the fixation of PHFs with interlocking intramedullary nails, Kirschner wires are often used to hold the bone fragments in a limited incision for closed reduction [14–16]. Herein, the minimally invasive procedure used in the intramedullary nail group resulted in reduced intraoperative blood loss compared to the larger incision size utilized in the locking plate group. Meanwhile, the surgery time was shorter in the locking plate group compared to the intramedullary nail group. The most time-consuming step in the intramedullary nail group was the reduction in the lesser or greater tuberosities for the three- and four-part type PHFs. During surgery, the greater and lesser tuberosities were manipulated and reduced using Kirschner wires as “joysticks” [17]. After the reduced fragments were temporarily fixed with Kirschner wire, a three- or four-part fracture becomes a simple two-part fracture.

Gardener first proposed medial support to avoid loss of the reduction [18]. Besides that, he proposed the concept of the humeral calcar screw: a long and inclined locking screw obliquely upward, precisely placed next to the inferior medial region of the humerus neck, in order to achieve stable medial support and reliable biomechanical stability. The Multiloc intramedullary nail adds to the calcar screw design to provide better support for patients with large proximal medial cortical defects [19]. Several previous studies demonstrated the biomechanical advantages of the intramedullary nail [14–16, 20]. There is a high risk of secondary displacement and fracture nonunion in elderly patients with poor bone quality. Studies have reported that bone grafting in the bone defect or intramedullary cavity before plate fixation can obtain good stability and clinical outcomes [18, 21, 22]. Lee et al. conducted a study to evaluate the clinical outcomes of locking plate fixation with a fibular strut allograft in the treatment of osteoporotic PHFs[23]. Importantly, the results showed that this strategy could significantly reduce the incidence of complications. In the locking plate group of our study, the bone defect of humeral head was evaluated during the operation, and autologous or allogeneic iliac bone grafting was used for patients with larger defects to increase the stability after plate fixation.

There are several limitations to our study. Firstly, this is a retrospective, non-randomized study and all the surgeries were performed by the same surgical team. Secondly, the bone mineral density of every patient was not evaluated. However, the mean age of these two groups showed no statistical significance. Thirdly, long-term complications such as humeral head necrosis might have been overlooked since the mean postoperative follow-up was only 9 months [24].

## Conclusions

The interlocking intramedullary nail approach is less invasive compared to the use of locking plates but takes longer to achieve fracture reduction and repair. There was no significant difference in pain and shoulder function scores between the two internal fixation approaches for the treatment of proximal humerus fractures. Detailed preoperative evaluation and accurate intraoperative operation, combined with the clinical experience of orthopedic surgeons, can improve the postoperative satisfaction of patients by designing individualized surgical plans and enhancing postoperative functional exercise guidance.

## Data Availability

All data generated or analyzed during this study are included in this published article.
